# Preliminary Characterization of Lignin-Modified Binder for Half-Warm-Mix Asphalt

**DOI:** 10.3390/polym17081019

**Published:** 2025-04-09

**Authors:** Ana M. Rodríguez Pasandín, Pablo Orosa Iglesias, Ignacio Pérez Pérez, Ana M. Rodríguez-Alloza

**Affiliations:** 1 School of Civil Engineering and Centro de Innovación Tecnolóxica en Edificación e Enxeñaría Civil (CITEEC), CGM, Universidade da Coruña, Campus de Elviña s/n, 15171 A Coruña, Spain; ignacio.perez1@udc.es; 2 Lyles School of Civil and Construction Engineering, Purdue University, West Lafayette, IN 47906, USA; porosaig@purdue.edu; 3 Departamento de Ingeniería Civil, Náutica y Marítima, Universidad de La Laguna, 38200 Santa Cruz de Tenerife, Spain; arodrial@ull.edu.es

**Keywords:** lignin, biobinder, half-warm-mix asphalt, microstructure, rheology

## Abstract

There is a growing trend to promote circular economy practices and reduce petroleum-derived product consumption in the paving sector. In this context, a liquid lignin-rich industrial waste was incorporated at 0% (control), 5%, 10%, 15%, and 20% into a bitumen emulsion to manufacture a lignin-based biobinder for half-warm-mix asphalt (HWMA). The mix of the bitumen emulsion and the industrial waste was made using an Ultra-turrax device, with the final mixing temperature monitored using a thermographic camera. Microstructure analysis was conducted using scanning electron microscopy (SEM). The bitumen was extracted and characterized using needle penetration tests at several temperatures. Additionally, the ring-and-ball softening point, penetration index, and ductility were assessed. Incorporating up to 5% of lignin-rich industrial waste led to a lignin-based biobinder that could be used for a more sustainable and bitumen-efficient HWMA production.

## 1. Introduction

Bitumen is a widely known byproduct of the petroleum industry. A reduced bitumen consumption in the paving sector is essential due to the global depletion of fossil fuels, the need to shift toward renewable energy sources [1], and the high cost of bitumen. Regarding the costs, according to national bitumen providers, the price of one ton of bitumen and one ton of polymer-modified bitumen, VAT excluded, was around 660 and 780 euros, respectively, in March 2025. Moreover, reduced bitumen consumption is also essential due to its harmful environmental impacts (such as gas emissions and related energy consumption) [2].

In this situation, lignin, a natural biopolymer, emerges as an opportunity. Many studies have looked into lignin’s potential as a bitumen extender or modifier [2,3,4] because of its cementitious qualities [5], abundant availability, and hydrocarbon-rich structure [6].

As shown in Table 1, most researchers found improved rutting resistance and reduced fatigue life when replacing different percentages of bitumen with lignin from different sources to manufacture bituminous mixtures. In this regard, Xu et al. [7] used 5% and 10% of commercial lignin powder from wood, Norgbey et al. [8] used 5% and 10% of waste lignin from bioethanol production, and Gao et al. [9] used 2%, 4%, 6%, and 8% commercial lignin powder from wood. Other authors found the same results using preheated lignin to remove the water content. This was the procedure followed by Ren et al. [10] and Yu et al. [11], who preheated the lignin at 100 °C for 2 h and 1.5 h, respectively. At this point, it is interesting to highlight that, conversely to the other authors, Yu et al. [11] also found improved fatigue resistance. Some researchers also found improved adhesive strength [10] and aging resistance [7]. Similarly, Nahar et al. [12] investigated chemically modified lignin alongside native lignin. These authors successfully substituted 25% of the weight of bitumen 70/100 with a commercial Kraft and Organosolv lignin. They stated that the compatibility of lignin modifications with bitumen depends critically on bitumen’s composition. That is, careful characterization of both bitumen and lignin should be conducted when using lignin-modified bitumen.

Finally, it is worth noting that other researchers only analyzed the suitability of using lignin as a partial substitute for bitumen in bituminous mixtures, primarily in hot-mix asphalt (HMA) applications. Arafat et al. [13] studied three types of lignin in percentages of 2%, 4%, and 6%, concluding the suitability of using up to 6% lignin to manufacture HMA. Zabelkin et al. [14] successfully used 5% of pyrolytic lignin for bituminous mixture manufacture. Similarly, Gaudenzi et al. [15] analyzed percentages of 30% of bitumen substitution.

Nevertheless, most studies have employed commercial lignin or lignin extracted from waste rather than utilizing lignin-rich waste in its untreated form. Only a few investigations have focused on directly using untreated lignin-rich waste materials [16,17,18,19]. As shown in Table 1, this previous research successfully used lignin-rich industrial waste from the hardboard manufacturing industry to modify a bitumen for hot-mix asphalt production or to modify an asphalt emulsion for half-warm-mix recycled asphalt production.

## 2. Aims and Scope

**Figure 1:**
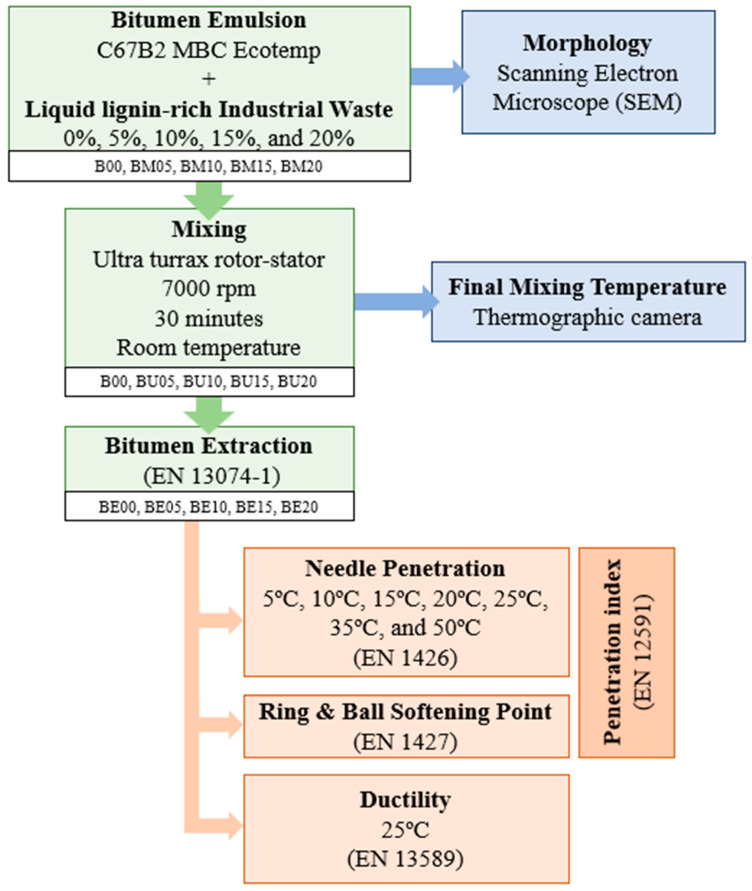
Flowchart of the research.

Given the need to advance more sustainable paving techniques and the growing scarcity of petroleum-based resources, like bitumen, this study aimed to characterize a biobinder composed of bitumen emulsion and an industrial lignin-rich waste from the hardboard manufacturing industry to assess its viability. As shown in the Introduction Section, biopolymer lignin has demonstrated its potential to be used as a bitumen extender or modifier. Thus, the use of this lignin-rich waste as a bitumen emulsion modifier could be of great interest to the paving sector and the wood industry. Lignin-based biobinder samples were prepared with varying lignin-rich waste content and analyzed according to the process outlined in the flowchart presented in Figure 1. Based on previous studies [16,17,18,19], varying contents of lignin-rich industrial waste, ranging from 5 to 20 percent, were used in this study to prepare the biobinder samples and compared with a control binder with 0 percent waste. The industrial waste was first manually mixed with the bitumen emulsion, and the resulting blend’s microstructure and morphology were assessed using scanning electron microscopy (SEM) techniques. To ensure thorough mixing of both liquids, the blending process was carried out using an Ultra-Turrax rotor/stator dispersing instrument at 7000 rpm for 30 min at room temperature (20 ± 5 °C) [16,17,18,19]. A thermographic imaging camera was used to monitor the final temperature of the blends, detecting any potential temperature increases during the mixing process. Finally, the binder from these samples was extracted according to the procedure outlined in the EN 13074-1 [20] standard. The extracted binders were then characterized using several tests: the needle penetration test (EN 1426 [21]) at temperatures ranging from 5 °C to 50 °C, the ring-and-ball softening point test (EN 1427 [22]), allowing to determine the penetration index (EN 12591 [23]), and the ductility test at 25 °C (EN 13589 [24]).

## 3. Materials and Methods

### 3.1. Commercial Bitumen Emulsion

The current study employed a commercial cationic bitumen emulsion, type C67B2MBC Ecotemp (Repsol, Madrid, Spain), designed for half-warm applications and supplied by a national company as needed. According to the manufacturer, its residual binder content was 65 to 69 percent, and its minimum fluidizing agent content was at least 2 percent. The control sample, totally composed of this commercial bitumen emulsion, was designed as B00.

### 3.2. Liquid Industrial Waste

**Figure 2:**
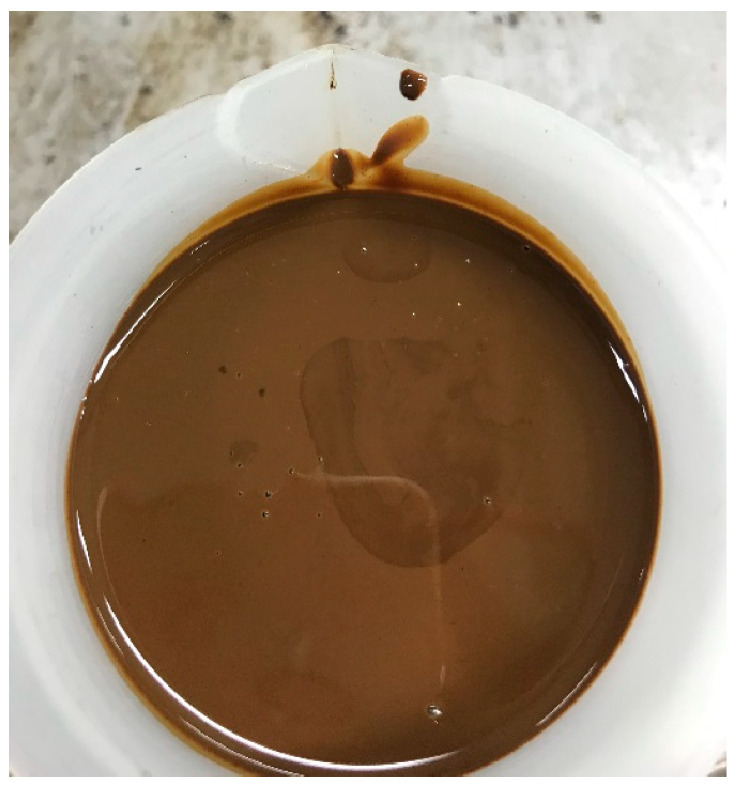
Brown and viscous liquid industrial waste.

The wood extracts produced during the production of high-density fiber boards by the wet process were the source of the industrial waste employed in this study (Figure 2). For the present preliminary characterization, this industrial waste was provided by a local supplier (Betanzos HB, Betanzos, Spain) on demand, according to the needs of the research. There would be no issues related to its use as a binder additive at an industrial scale because the generation of this waste at the plant is high. According to the supplier, its plant alone generates 8000 tons/year of waste from producing approximately 70,000 m^3^ of wood panels. The statistical data from the Food and Agricultural Organization (FAO) of the United Nations show that Germany and Poland are the major European wood panel producers [25]. These two countries alone host at least 18 plants operating with the same production process [26].

This wood extract is a co-product made entirely of natural ingredients because no artificial glues for bonding fibers are added to the manufacturing process. These compounds are also high-value-added ingredients, like sugars, tannins, polyphenols, and lignin. Because the supplier mainly uses eucalyptus, a variation in the co-product composition is not expected. Table 2 displays its key features and composition, as stated by the supplier.

It is worth noting that the waste is a byproduct, as it is generated at the same time as the production of the wood panels. The production uses renewable raw materials from sustainably managed sources, certified sustainable biomass energy, and no chemical additives. All of this guarantees the sustainability of the waste used. In line with sustainability, the waste was used as is, i.e., without any drying or treatment.

### 3.3. Mixing Process

In the present research, five liquid industrial waste substitution percentages were tested: 0 (control), 5, 10, 15, and 20 percent in mass of the bitumen emulsion. First, the C67B2MBC Ecotemp bitumen emulsion and the adequate amount of liquid industrial waste were measured and hand-mixed using a glass stirrer. To identify the industrial waste content of the hand-mixed blends, they were named BM05, BM10, BM15, and BM20, with the numbering indicating the waste percentage substitutions indicated before. Then, an IKA T-25 Ultra-turrax digital disperser (IKA Works Spain, Barcelona, Spain) provided with a stainless-steel dispersion tool (rotor/stator with slots) was used to complete the blend. After several trials, the bitumen emulsion and the liquid industrial waste were mixed by a 30 min mixing at 7000 rpm using this mixing device. At the beginning of the mixing process with the Ultra-turrax device, the blend was at room temperature (20 ± 5 °C). The blends mixed with the Ultra-turrax device were designated as BU05, BU10, BU15, and BU20, corresponding to the percentage of waste substitution used in the previous nomenclature.

### 3.4. Laboratory Analysis

#### 3.4.1. Microstructure Analysis

SEM (scanning electron microscope) techniques were used to analyze the blend’s microstructure and morphology before using the Ultra-turrax device. Percentages of substitution of bitumen emulsion by the liquid industrial waste of 0 (control), 5, 10, 15, and 20 percent were examined before using the Ultra-turrax (B00, BM05, BM10, BM15, and BM20). For this purpose, a JEOL JSM-6400 microscope (Jeol, Tokyo, Japan) was used. This microscope launches an electron beam using a thermionic electron gun with a tungsten filament. The electron beam travels across the sample’s surface and interacts with the atoms that make up the sample, generating various signals picked up by detectors. This equipment includes the detectors for backscattered electrons (resolution of 10 nm at an 8 mm working distance) and secondary electrons (at 25 kV, resolution of 3.5 nm at an 8 mm working distance and 10 nm at a 39 mm working distance) as well.

#### 3.4.2. Temperature Monitoring

**Figure 3:**
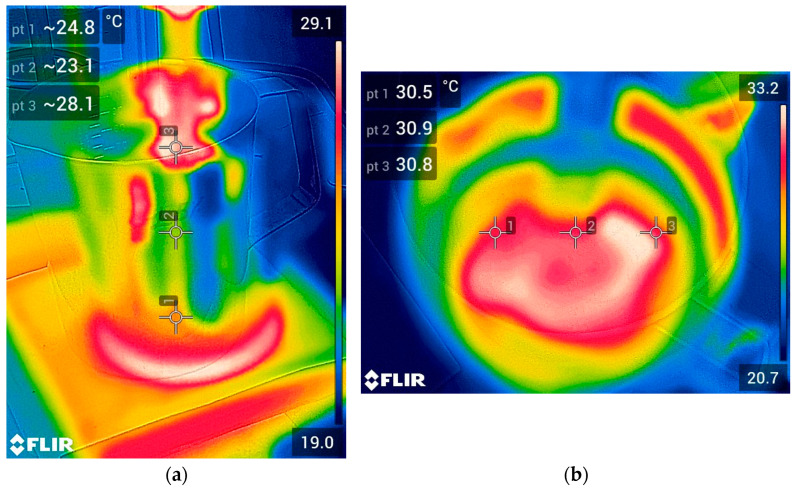
Thermographic picture of the blend of bitumen emulsion and 15% industrial waste: (**a**) at the beginning of the mixing process and (**b**) at the end with the Ultra-turrax device.

During the blending of both liquids, i.e., the bitumen emulsion and the industrial waste, using the Ultra-turrax device, a rather pronounced warming of the resulting biobinder was observed. For this reason, a thermographic imaging camera, model FLIR E53 (Teledyne FLIR, Wilsonville, OR, USA), which can record infrared radiation and convert it into pictures that correspond to various temperature readings, was used to determine the final temperature of the BU05, BU10, BU15, and BU20 after 30 min of mixing using the Ultra-turrax device (Figure 3). The temperature measurements were conducted using a thermographic camera each time the blend was prepared (on different days) to ensure the consistency of the results.

#### 3.4.3. Binder Extraction

**Figure 4:**
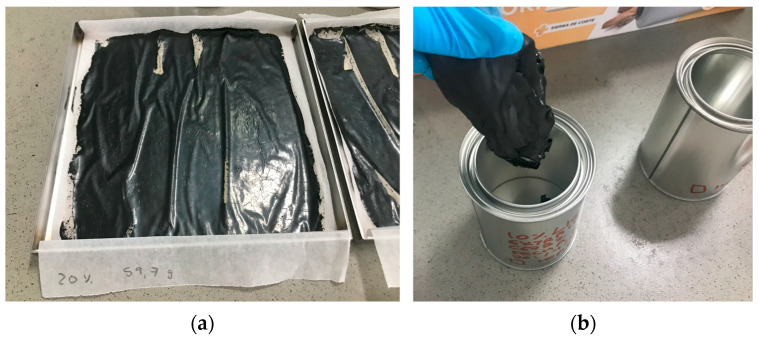
Details of the binder extraction procedure: (**a**) binder (in this case, bitumen with 20% of industrial waste) placed in a thin layer over a tray and (**b**) recovered binder (in this case, bitumen with 10% of industrial waste) being introduced in a can.

EN 13074-1 [20] describes the procedure followed in the present research to extract the residual binder (bitumen and industrial waste) from the bitumen emulsion. This process involves applying a thin layer of bitumen emulsion to a tray (Figure 4a). After 24 h at room temperature, the bitumen emulsion is subjected to another period of 24 h, but, in this case, at 50 °C in a ventilated oven. Then, the residual binder can be recovered (Figure 4b). Following the previous nomenclature, the samples of the recovered binder were named BE00, BE05, BE10, BE15, and BE20, with the numbering indicating the waste percentage substitution, as explained before.

#### 3.4.4. Ductility

**Figure 5:**
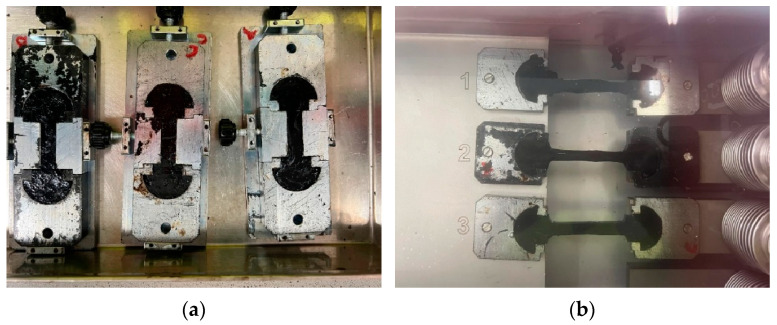
Ductility test: (**a**) sample (BE00) preparation and (**b**) samples (BE00) at the beginning of the test, in the ductilometer.

The ultimate goal of the ductility analysis was to determine the tensile properties of the recovered binder (BE00, BE05, BE10, BE15, and BE20) following the test described in EN-13589 [24]. For each of the recovered binders, a total of three samples were tested. First, three standardized molds (Figure 5a) were filled with the recovered binder and left at room temperature for 1.5 ± 0.5 h. Then, the molds were leveled with a hot spatula and placed in a water bath at the test temperature (5 ± 0.5 °C) for 90 ± 10 min. After this time, they were partially removed from the mold and put in the ductilometer. Finally, these samples were stretched at a constant speed of 50 ± 2.5 mm/min at the test temperature until an elongation of 0.4 m was achieved (Figure 5b).

#### 3.4.5. Needle Penetration

The bitumen penetration grade was determined according to EN 1426 [21]. In this test, the penetration in tenths of a millimeter was determined to identify the consistency of the binder (BE00, BE05, BE10, BE15, and BE20). For this purpose, a needle with a standard load of 100 g was applied to the binder sample for 5 s at 25 °C, with the result being the average of a total of three determinations on the same recovered binder sample. In this research, in addition to 25 °C, the needle penetration test was conducted at other temperatures: 5 °C, 10 °C, 15 °C, 20 °C, 30 °C, 35 °C, 40 °C, 45 °C, and 50 °C.

#### 3.4.6. Ring-And-Ball Softening Point

The EN 1427 was followed to measure the ring-and-ball softening point [22]. The test involved heating a total of two horizontal discs of the recovered binder (BE00, BE05, BE10, BE15, and BE20), each supporting a steel ball, at a controlled rate of 5 °C per minute in a water bath. The discs were cast in brass rings. The softening point was the average temperature at which the two discs softened to the point where each ball covered in bituminous binder could fall 25.0 ± 0.4 mm.

#### 3.4.7. Penetration Index

This index gives an idea of the thermal susceptibility of the bituminous binders. It was calculated as indicated in the EN 12591 standard [23]:

Ip = (20 tRaB + 500 logP − 1952)/(tRaB − 50 logP + 120),(1)

where tRaB is the softening point (°C), and P is the penetration at 25 °C (in 0.1 mm).

## 4. Results and Discussion

### 4.1. Microstructure Analysis

**Figure 6:**
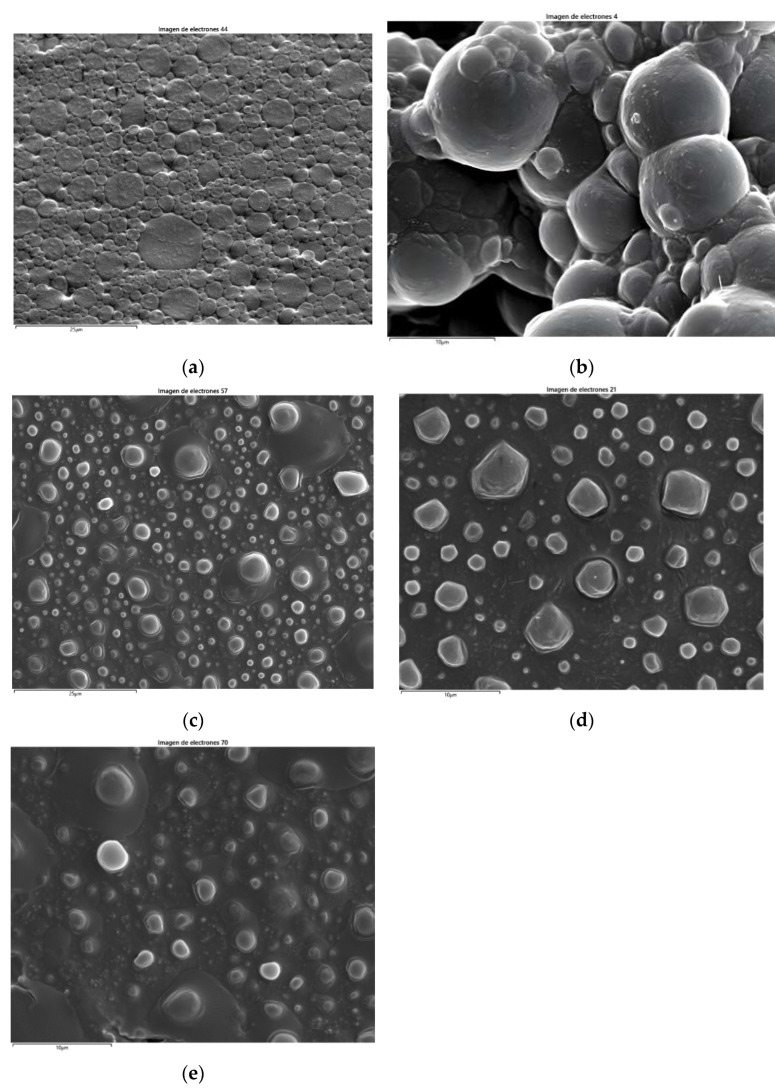
SEM images on the same scale of (**a**) B00, (**b**) BM05, (**c**) BM10, (**d**) BM15, and (**e**) BM20.

SEM techniques were used on the blends of bitumen emulsion and liquid lignin-rich waste before using the Ultra-turrax mixing device to assess its microstructure and morphology. The SEM’s most representative images of the blends with different percentages of liquid waste are shown in Figure 6. At an industrial level, the most used bitumen emulsions for road pavement applications have a bitumen content that ranges from 48% to 62% [27], or even up to 70% [28], and the micelles’ sizes from 1 to 10 μm [29]. As shown in Figure 6a, the B00 samples presented the typical bitumen emulsion microstructure with spherical micelles, with sizes in the micrometric order, within the range indicated above. However, as increasing amounts of industrial waste were added and more vigorous manual mixing was required to achieve a homogeneous mixture, a change in the microstructure of the final product could be seen. In this regard, in BM05, the spherical micelles of bitumen, with different sizes (in the usual range of 1 to 10 μm), could still be perceived. However, clear signs of agglomeration and flocculation were also present, indicating the early stages of bitumen emulsion breaking. The microstructure changes from BM10 to BM20 showed loose particles floating in a continuous medium, in most cases with irregular shapes instead of spherical shapes. This indicates that higher industrial waste contents and increased mixing energy contributed to the disappearance of bitumen micelles. Also, as the waste content increased, the bitumen content in the bitumen emulsion decreased. In this regard, introducing 20% waste led to bitumen emulsions with 50% to 55% of bitumen content, which is within the usual range (48% to 70%).

It is evident from the particle size and shape that no sign of the usual bituminous emulsion microstructure was left behind when residue concentrations exceeded 5%.

Nevertheless, regarding bitumen content, all the tested waste percentages were in the usual range.

### 4.2. Temperature Monitoring

**Figure 7:**
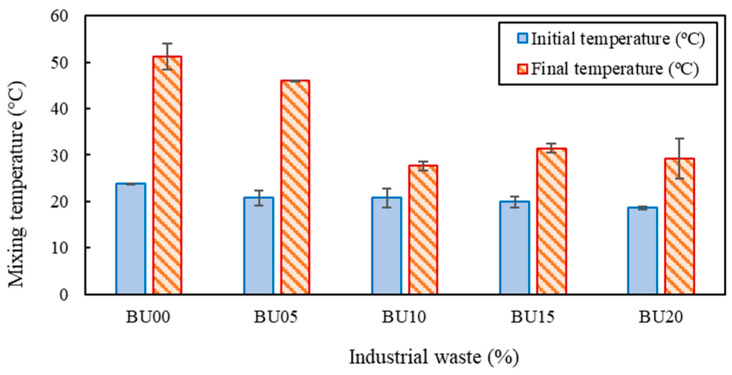
Initial and final samples’ temperatures when mixed using the Ultra-turrax device.

Figure 7 shows the results obtained from the thermographic images of BU00, BU05, BU10, BU15, and BU20 at the beginning and the end of the mixing process using the Ultra-turrax device. Because the room temperature differed when the measurements were taken, there were minor variations in the samples’ initial temperatures, as seen in Figure 7. When only the bitumen emulsion (BU00) was subjected to the high-speed mixer, the final temperature of the sample was over 50 °C. When a 5% industrial waste was added (BU05), the final temperature was lower but was still high (over 45 °C). Nevertheless, for the increased industrial waste contents (BU10, BU15, and BU20), the final temperature was around 30 °C. This effect could be explained by the water content in the industrial waste, which helps to fluidize the mixture and, therefore, reduce internal friction that leads to a decrease in temperature. It is interesting to note that all the final temperatures were below 80 °C, which is the maximum allowable heating temperature [30] for the bitumen emulsion, to avoid its premature breaking. For this reason, in terms of heating temperature, all the tested percentages could be used at the industrial level.

### 4.3. Ductility, Needle Penetration, Softening Point, and Penetration Index

The binder from the biobinders was extracted for characterization after they were mixed using the Ultra-turrax device. The characterization included ductility, needle penetration, ring-and-ball tests, and the determination of the penetration index. Table 3 summarizes the results from the ductility and ring-and-ball tests and the penetration index obtained for the binders extracted from the prepared biobinder.

The ductility results in Table 3 demonstrate that as the industrial waste percentage increased, the force required to accomplish 0.400 m of elongation generally rose as well, resulting, as was expected, in decreased ductility. The chemical nature of the industrial waste dry matter, which included 41.6% sugar and 23.4% lignin, among other compounds (Table 2), may contribute to this performance. Other researchers [8,11] found reduced ductility as the lignin content increased, which is also in line with the stiffening effect of the lignin found by several authors [7,8,9,12]. It is important to note that while this represents a general trend, the BE15 sample did not conform to it. This deviation was likely due to the heterogeneities introduced by the industrial waste. Despite the blend of the bitumen emulsion and the waste being made under controlled conditions (time and speed), the final microstructure was not homogeneous (Figure 6) due to the nature of the waste. It should be noted that there are no specific limits for the ductility force values in the current European specifications [31] for the bitumen extracted from a bitumen emulsion to be used at the industrial level. In addition, the ring-and-ball softening point results shown in Table 3 for the different extracted biobinders were quite similar across all samples, although a slight increase was observed as the residue content increased. This increase was particularly noticeable in the case of BE20 due to its higher waste content. Other researchers found an increase in the softening point with increased lignin contents [8,11], which again aligned with the stiffening effect of the lignin found by several authors [7,8,9,12].

**Figure 8:**
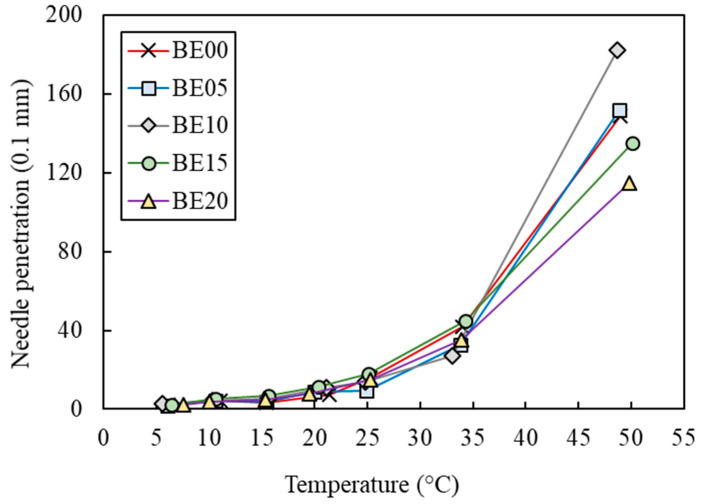
Needle penetration results of the extracted binders at different temperatures.

Figure 8 allows us to assess the evolution of the needle penetration of the extracted biobinders (BE00, BE05, BE10, BE15, and BE20) with the temperature. It can be appreciated that the higher the temperature, the higher the differences in penetrations between samples. It was an expected result due to the higher stiffness of the bitumen at low temperatures. Nevertheless, it can be seen that the results of the BE00 were very similar to that of the BE05. Also, it is interesting to note that, in most cases, the samples displayed higher penetrations when the waste content was lower. However, similar to the previous section, all the waste contents did not follow this trend. Remarkably, BE10 did not follow this trend. Again, the heterogeneities introduced by the liquid waste seemed to be mainly responsible for this performance. Table 4 shows the standard deviations.

Considering the European technical specifications [31] for the extracted bitumen used for bituminous emulsion manufacturing, the ring-and-ball values and the penetration values at 25 °C were consistent with the specified limits for bituminous emulsion classes 4 (penetration ≤ 150 0.1 mm and ring and ball ≥ 50 °C), 5 (penetration ≤ 220 0.1 mm and ring and ball ≥ 46 °C), 6 (penetration ≤ 270 0.1 mm and ring and ball ≥ 43 °C), and 7 (penetration ≤ 330 0.1 mm and ring and ball ≥ 39 °C).

Finally, the penetration index results in Table 3 show that the Ip of the samples BE00 to BE15 were very similar. Nevertheless, the BE20 displayed significantly higher Ip. In general, higher Ip is associated with lower thermal susceptibility. However, again, this result could be due to heterogeneities introduced by the industrial waste. It should be noted that there are no specific limits for the Ip values in the current European specifications [31] for bitumen extracted from a bitumen emulsion to be used at the industrial level.

As can be seen, the substitution of bitumen emulsion by liquid industrial waste rich in lignin biopolymer introduced heterogeneities, particularly when the substitution percentage was higher than 5%. Nevertheless, using this waste up to 5% led to biobinders that performed similarly to the control binder (C67B2 MBC Ecotemp). This outcome is in line with the observed microstructure of the blends of bitumen emulsion and industrial waste in the SEM images (Figure 6). Percentages of industrial waste from 10% to 20% led to a microstructure that totally differed from the microstructure of the original bitumen emulsion, while using only 5% of industrial waste maintained it, despite that certain agglomeration and flocculation were detected at the microstructural level.

## 5. Conclusions

This study aimed to preliminarily characterize the biobinders obtained when mixing increased amounts of a liquid industrial waste rich in lignin biopolymer with cationic bitumen emulsion for HWMA production. In this regard, percentages of 0% (control), 5%, 10%, 15%, and 20% of the industrial waste were analyzed. The following conclusions and findings were obtained:

- As expected, the inclusion of industrial waste introduced heterogeneities that could explain the lack of a clear trend in the performance of the biobinders with the increased amount of waste. Nevertheless, despite it being an expected result, the substitution percentage from which the heterogeneities will appear was unknown before conducting the present preliminary characterization.
- Nevertheless, the microstructures of the B00 and the BM05 samples were very similar, which reinforces the idea that the maximum replacement percentage is around 5%.
- Moreover, when using the Ultra-turrax device, the final temperatures of the BU00 and the BU05 were higher than those of the other samples.
- In addition, the needle penetration results were very similar for the BE00 and the BE05.

Despite the heterogeneities, it can be stated that the substitution of 5% of cationic bitumen emulsion by liquid industrial waste led to biobinders with similar performance to that of the control binder (0% industrial waste). This suggests that incorporating up to 5% of lignin-rich liquid industrial waste leads to a lignin-based biobinder that could be used for more sustainable and bitumen-efficient HWMA for road paving. However, a comprehensive rheological characterization should be conducted, including DSR, BBR, penetration, and ring-and-ball tests before and after RTFOT and/or PAV aging. These evaluations should include the 5% substitution level along with nearby percentages (e.g., 3%, 4%, 6%, and 7%) to better understand the performance trends and identify the optimal substitution rate.

## Figures and Tables

**Table 1:** Summary of the main findings according to the technical literature review.

Reference	Lignin Source	Lignin Proportion in Bitumen (%)	Main Findings
[7]	Commercial wood lignin powder	5% and 10%	Rheology of modified bitumen was conducted. Increased lignin content increased rutting resistance (15% to 50% increase in the rutting parameter). The higher the lignin content, the lower the fatigue resistance. Improved aging resistance due to reduced formation of carbonyl groups. Stiffening effect of the lignin.
[8]	Waste lignin from the production of bioethanol from corncob	5% and 10%	Rheology of modified bitumen was conducted. The higher the lignin content, the higher the rutting resistance and the lower the fatigue resistance. Stiffening effect of lignin. Higher ductility force (reduced ductility), lower penetration, and higher ring-and-ball temperature as lignin content increases.
[9]	Commercial wood lignin powder	2%, 4%, 6%, and 8%	Rheology of modified bitumen was conducted. Increased lignin content increased rutting resistance (12% to 68% increase in the rutting parameter). Small reduction in fatigue resistance. Stiffening effect of the lignin.
[10]	Source of lignin not specified (preheated)	10%, 20%, and 30%	Rheology of modified bitumen was conducted. The higher the lignin content, the higher the rutting resistance and the lower the fatigue resistance. Improved adhesive strength.
[11]	Commercial soda lignin powder (preheated)	5%, 10%, 15%, and 20%	Rheology of modified bitumen was conducted. The higher the lignin content was, the higher the rutting resistance. Improved fatigue resistance when using 5% and 10% of lignin (increased number of cycles to failure ranging from 1.6 to 2). Increased lignin contents led to reduced ductility, lower penetration, higher ring-and-ball temperature, and higher penetration index.
[12]	Commercial kraft and Organosolv (chemically modified and native)	25%	Rheology of modified bitumen was conducted. Viscoelastic performance of lignin-modified bitumen was similar to that of the base bitumen. Higher stiffness of the lignin-modified bitumen. Compatibility between lignin and bitumen is highly dependent on their type and composition. Stiffening effect of the lignin.
[13]	Commercial kraft lignin, lignin precipitated from black liquor, and lignin produced from rice hulls	2%, 4%, and 6%	Higher rutting resistance of HMA (14% less rut depth when using 6% lignin from black liquor) and adequate moisture damage resistance.
[14]	Pyrolytic lignin	5%, 10%, 20%, and 50%	Increased compressive strength of HMA mixtures (up to 3.76 times higher when 10% lignin was used). Increased moisture damage resistance when using up to 20% lignin. Using up to 20% of lignin led to HMA mixtures complying with Russian standards.
[15]	Powder lignin from thermochemical and enzymatic treatments of wood (dried)	30%	Lower fatigue resistance of HMA mixtures made with lignin.
[16]	Lignin-rich industrial waste	5%, 10%, 20%, and 40%	The foaming effect of the liquid waste, when blended with the base bitumen, improves the HMA blending. Improved water resistance of HMA made with 20% lignin as a substitution for bitumen.
[17]		5%, 10%, 20%, and 40%	After a rheological characterization of the blend of bitumen and waste, the use of 20% waste showed the best potential to be used as a bitumen extender.
[18]		5%, 10%, 15%, and 20%	Half-warm-mix asphalt made with 100% reclaimed asphalt pavement, using bitumen emulsion partially substituted by 5% of the waste, displayed slightly higher water resistance (tensile strength ratio 1.35% higher).
[19]		5%, 10%, 15%, and 20%	The results showed that using blends of asphalt emulsion and waste lignin for half-warm-mix asphalt made with 100% reclaimed asphalt pavement up to 15% improves the mixture’s cohesion, but only substitutions up to 5% result in mixes with improved water resistance. According to the life cycle assessment, as the percentage of substitution of bitumen emulsion by waste lignin grows, the greater the CO_2_e savings.

**Table 2:** Composition and main properties of the liquid lignin-rich industrial waste.

**Composition**	**Water**	56.31%	
	**Dry matter**	43.69%	
		Sugar	41.6%
		Lignin Insoluble Klason lignin (16.3%)	23.4%
		Soluble lignin (7.1%)	
		Pectin	13.3%
		Polyphenols	11.8%
		Mineral matter	9.5%
		Other compounds	0.4%
**C, H, O, N, S analysis**		C: 46%, H: 5.8%, S: 0.07%	
**pH**	3.2		
**Density (at 75 °C)**	1155 kg/m^3^		
**Viscosity (at 80 °C)**	14 mPa·s		

**Table 3:** Summary of the results from the ductilometer (force applied to achieve 0.400 m of elongation), softening point, and penetration index for the extracted binders.

Sample	Ductilometer	Ring and Ball	Penetration Index
	Force, N (Standard Deviation)	Softening Point, °C (Standard Deviation)	Ip
BE00	0.66 (0.026)	51.9 (1.27)	−2.8
BE05	0.88 (0.041)	52.9 (0.49)	−3.3
BE10	0.97 (0.102)	53.6 (1.27)	−2.7
BE15	3.38 (0.702)	51.7 (0.00)	−2.7
BE20	1.27 (0.019)	62.7 (0.35)	−0.9

**Table 4:** Standard deviations (needle penetration test results).

Sample/Temperature	5 °C	10 °C	15 °C	20 °C	25 °C	35 °C	50 °C
BE00	0.050	0.332	0.078	0.033	0.245	0.337	2.563
BE05	0.031	0.014	0.138	0.156	0.196	0.246	1.013
BE10	0.093	0.085	0.109	0.104	0.111	0.198	0.444
BE15	0.041	0.021	0.096	0.123	0.322	0.265	1.627
BE20	0.080	0.057	0.021	0.090	0.094	0.161	0.344

## Data Availability

The data are available upon request. The data presented in this study are available on request from the corresponding author. The data are not publicly available due to the research is still ongoing.

## References

[1] IRENA (2024). 100% Renewable Energy Scenarios Supporting Ambitious Policy.

[2] Gaudenzi E., Cardone F., Lu X., Canestrari F. (2023). The use of lignin for sustainable asphalt pavements: A literature review. Constr. Build. Mater..

[3] Yatish R.G., Kumar D.H., Chinnabhandar R.K., Raviraj H.M., Shankar A.R. (2024). A review of the potential application of lignin in the production of bio-binder: Challenges and opportunities. J. Mater. Sci..

[4] Yao H., Wang Y., Liu J., Xu M., Ma P., Ji J., You Z. (2022). Review on applications of lignin in pavement engineering: A recent survey. Front. Mater..

[5] Luz P.M.S.G., Ziegler C.R., Mendonça A.M.G.D., Rodrigues J.K.G. (2021). Rheological evaluation of pg 64–122 asphalt binder modified with lignin of pinus and eucalyptus woods. Mater. Struct..

[6] Brauns F.E. (1951). The Chemistry of Lignin.

[7] Xu G., Wang H., Zhu H. (2017). Rheological properties and anti-aging performance of asphalt binder modified with wood lignin. Constr. Build. Mater..

[8] Norgbey E., Huang J., Hirsch V., Liu W.J., Wang M., Ripke O., Nkrumah P.N. (2020). Unravelling the efficient use of waste lignin as a bitumen modifier for sustainable roads. Constr. Build. Mater..

[9] Gao J., Wang H., Liu C., Ge D., You Z., Yu M. (2020). High-temperature rheological behavior and fatigue performance of lignin modified asphalt binder. Constr. Build. Mater..

[10] Ren S., Liu X., Zhang Y., Lin P., Apostolidis P., Erkens S., Xu J. (2021). Multi-scale characterization of lignin modified bitumen using experimental and molecular dynamics simulation methods. Constr. Build. Mater..

[11] Yu J., Vaidya M., Su G., Adhikari S., Korolev E., Shekhovtsova S. (2021). Experimental study of soda lignin powder as an asphalt modifier for a sustainable pavement material. Constr. Build. Mater..

[12] Nahar S., Slaghek T.M., van Vliet D., Haaksman I.K., Gosselink R.J. (2023). Mutual compatibility aspects and rhe-ological assessment of (modified) lignin–bitumen blends as potential binders for asphalt. Road Mater. Pavement Des..

[13] Arafat S., Kumar N., Wasiuddin N.M., Owhe E.O., Lynam J.G. (2019). Sustainable lignin to enhance asphalt binder oxidative aging properties and mix properties. J. Clean. Prod..

[14] Zabelkin S., Bikbulatova G., Grachev A., Bashkirov V., Burenkov S., Makarov A. (2019). Modification of bitumen binder by the liquid products of wood fast pyrolysis. Road Mater. Pavement Des..

[15] Gaudenzi E., Ingrassia L.P., Cardone F., Lu X., Canestrari F. (2023). Investigation of unaged and long-term aged bio-based asphalt mixtures containing lignin according to the VECD theory. Mater. Struct..

[16] Pérez I.P., Pasandín A.M.R., Pais J.C., Pereira P.A.A. (2019). Use of lignin biopolymer from industrial waste as bitumen extender for asphalt mixtures. J. Clean. Prod..

[17] Pérez I., Pasandín A.R., Pais J.C., Pereira P.A. (2020). Feasibility of using a lignin-containing waste in asphalt binders. Waste Biomass Valorization.

[18] Pasandín A.R., Nardi E., Pérez-Barge N., Toraldo E. (2022). Valorisation of lignin-rich industrial byproduct into half-warm mix reclaimed asphalt with enhanced performance. Constr. Build. Mater..

[19] Rodríguez Pasandín A.M., Orosa P., Rodríguez-Alloza A.M., Nardi E., Pérez-Barge N. (2025). Effects of Untreated Waste Lignin as a Sustainable Asphalt Emulsion Substitute on Water Resistance and Environmental Impacts in Reclaimed Half-Warm Asphalt Mixtures. Coatings.

[20] (2019). Bitumen and Bituminous Binders. Recovery of Binder from Bituminous Emulsion or Cut-Back or Fluxed Bituminous Binders. Recovery by Evaporation.

[21] (2016). Bitumen and Bituminous Binders. Determination of Needle Penetration.

[22] (2015). Bitumen and Bituminous Binders. Determination of the Softening Point. Ring and Ball Method.

[23] (2009). Bitumen and Bituminous Binders. Specifications for Paving Grade Bitumens.

[24] (2019). Bitumen and Bituminous Binders. Determination of the Tensile Properties of Modified Bitumen by the Force Ductility Method.

[25] ForestProductStatistics Data|Forest Products Statistics|Food and Agriculture Organization of the United Nations. https://www.fao.org/forestry/statistics/data/en.

[26] European Panel Federation Producers—European Panel Federation. European Panel Federation. 2023. https://europanels.org/the-wood-based-panel-industry/producers/.

[27] Spanish Ministry of Public Works (2018). General Technical Specifications for Road and Bridge Works. Article 214. Bitumen Emulsions.

[28] Gorman J.L., Crawford R.J., Harding I.H. (2004). Bitumen emulsions in road construction—A review. Road Transp. Res..

[29] Ignatavicius S., Kavanagh A., Colleran D., Brennan M., Newell S. The use Anionic Bitumen Emulsions in Pavements—A state of the art review. Proceedings of the 7th Eurasphalt and Eurobitume Congress.

[30] Technical Association of Bituminous Emulsions, ATEB Half-Warm Mix Asphalt with Bitumen Emulsion. Report. 2014. https://www.ateb.es/images/pdf/monografias/Monografia%20Templadas.pdf.

[31] (2005). Bitumen and Bituminous Binders. Framework for Specifying Cationic Bituminous Emulsions.

